# CNS glucose metabolism in Amyotrophic Lateral Sclerosis: a therapeutic target?

**DOI:** 10.1186/s13578-020-00511-2

**Published:** 2021-01-11

**Authors:** Tesfaye Wolde Tefera, Frederik J. Steyn, Shyuan T. Ngo, Karin Borges

**Affiliations:** 1 Australian Institute for Bioengineering and Nanotechnology, The University of Queensland, Brisbane, QLD 4072 Australia; 2 School of Biomedical Sciences, The University of Queensland, Brisbane, QLD 4072 Australia; 3 Center for Clinical Research, The University of Queensland, Brisbane, Australia

**Keywords:** Amyotrophic lateral sclerosis, Brain energy metabolism, Glucose metabolism, Glycolysis, Pentose phosphate pathway, TCA cycle, Mitochondrial dysfunction, Neurodegeneration, Neuro-glial interactions, Motor neuron disease

## Abstract

Amyotrophic lateral sclerosis (ALS) is a fatal progressive neurodegenerative disorder primarily characterized by selective degeneration of both the upper motor neurons in the brain and lower motor neurons in the brain stem and the spinal cord. The exact mechanism for the selective death of neurons is unknown. A growing body of evidence demonstrates abnormalities in energy metabolism at the cellular and whole-body level in animal models and in people living with ALS. Many patients with ALS exhibit metabolic changes such as hypermetabolism and body weight loss. Despite these whole-body metabolic changes being observed in patients with ALS, the origin of metabolic dysregulation remains to be fully elucidated. A number of pre-clinical studies indicate that underlying bioenergetic impairments at the cellular level may contribute to metabolic dysfunctions in ALS. In particular, defects in CNS glucose transport and metabolism appear to lead to reduced mitochondrial energy generation and increased oxidative stress, which seem to contribute to disease progression in ALS. Here, we review the current knowledge and understanding regarding dysfunctions in CNS glucose metabolism in ALS focusing on metabolic impairments in glucose transport, glycolysis, pentose phosphate pathway, TCA cycle and oxidative phosphorylation. We also summarize disturbances found in glycogen metabolism and neuroglial metabolic interactions. Finally, we discuss options for future investigations into how metabolic impairments can be modified to slow disease progression in ALS. These investigations are imperative for understanding the underlying causes of metabolic dysfunction and subsequent neurodegeneration, and to also reveal new therapeutic strategies in ALS.

## Background

Amyotrophic Lateral Sclerosis (ALS) is a fatal progressive neurodegenerative disorder primarily characterized by selective degeneration of both the upper motor neurons in the brain and lower motor neurons in the brain stem and the spinal cord [1]. The death of these motor neurons leads to muscle weakness, paralysis and finally death primarily due to the loss of respiratory function [2]. Most forms of ALS start between 50 to 60 years of age and average life expectancy is 3 to 5 years after the onset of symptoms [1, 3]. However, in a small number of patients the disease progresses slowly [3]. Currently there are no effective treatments that can slow disease progression or cure ALS.

The majority (90%) of ALS cases do not have a single genetic origin (sporadic ALS), while 10% are familial [4]. More than 50 causative genes have been identified in ALS [5]. Among those, key mutations in genes such as superoxide dismutase 1 (SOD1) [6], TAR DNA-Binding Protein (TDP-43) [7], fused in sarcoma (FUS) [8] and chromosome 9 open reading frame 72 (C9ORF72) [9, 10] have been identified. Mutations in C9ORF72 are representative in 40% of familial ALS cases [2]. Twenty percent of familial ALS cases are linked to mutations in the SOD1 gene and this accounts for 1 to 2% of all forms of ALS, while FUS and TDP-43 mutations constitute 5% of familial ALS cases [4, 11]. Although the cause of the majority of sporadic ALS is unknown, recent studies have also reported familial ALS associated gene mutations in patients with sporadic ALS (without family history). Notably, mutations in C9OR72 gene constitute about 5% of sporadic ALS cases with European ancestry [12]. The number of genes identified to be associated with ALS is increasing*.* With the advancements in genetic analysis technologies, it is expected that other new genes will be discovered in the future [13].

The exact mechanisms underlying the selective death of motor neurons in ALS are not yet completely known. However, it is believed that the pathogenic processes in this disease are multifactorial and may not be mutually exclusive [14]. Major hypothesized mechanisms include glutamate excitotoxicity [15], abnormal protein aggregation [6, 16], impaired axonal transport [17], inflammation [18], oxidative stress [19], and abnormalities in energy metabolism [20, 21]. ALS is a complex disease where several pathological mechanisms contribute to neuronal loss. There is growing evidence that demonstrates a key role of dysfunctional energy metabolism in driving the onset and progression of ALS, which we and others have previously reviewed [22–24]. It is not yet clear whether metabolic abnormalities are the underlying cause of neuronal death or a consequence of disease. While some studies indicate that metabolic disruptions may occur before the loss of motor neurons, and long before the onset of motor symptoms [25–29], others have suggested the opposite [30]. However, it is likely that a combination of proposed ALS disease mechanisms, together with abnormal energy metabolism, could be involved in initiating and driving the selective death of motor neurons and denervation of muscle in ALS.

In this review, we summarize central metabolic abnormalities in ALS, particularly in the context of changes in glucose transport and metabolism in the CNS, which could contribute to the onset and progression of disease. First, we briefly discuss glucose metabolism in the CNS. Then, we focus on whole body metabolic changes in patients with ALS, and metabolic defects described in CNS glucose metabolism (glucose transport, glycolysis, pentose phosphate pathway (PPP), tricarboxylic acid (TCA) cycle, mitochondrial oxidative phosphorylation) in mouse and cellular models of ALS, as well as patients with ALS. We will also briefly highlight efforts to correct metabolic abnormalities with the aim of delaying the onset and progression of disease and improving survival. Finally, we briefly describe areas of metabolism research that needs further investigation.

## A brief overview of CNS glucose metabolism

Substantial amounts of energy are required to maintain basic physiological functions in the CNS [31]. The adult human brain constitutes approximately 2% of the total body weight, however, it consumes about 20% of the body’s energy. Most of the energy is used for maintaining action potentials and postsynaptic signaling [32, 33]. Neurons require a significant amount of energy and typically need a continuous availability of glucose from the blood, as stored energy in the brain is low [34]. This high energy demand is mostly met by oxidation of glucose via mitochondrial oxidative phosphorylation (OXPHOS). Under normal physiological conditions, glucose is the main obligatory energy substrate in the brain. However, when glucose availability is low such as during excessive physical activity, development, or prolonged starvation, it can be supplemented with other substrates, such as lactate, ketone bodies, and medium chain fatty acids [35–39].

The carbons derived from glucose are also used for the synthesis of lipids, amino acids and neurotransmitters in the brain, such as glutamate, γ-aminobutyric acid (GABA), glutamine and aspartate. Glucose is also important in the defense against oxidative stress as it is metabolized via the pentose phosphate pathway (PPP) to produce nicotinamide adenine dinucleotide phosphate (NADPH), which is needed to keep glutathione in its antioxidant reduced state [40]. Glucose moves across the endothelial membrane into the extracellular fluid via facilitated transport by glucose transporter 1 (GLUT1) [41]. GLUT1 also transports glucose from the extracellular fluid into astrocytes. In neurons, GLUT3 is the main glucose transporter and has a higher rate of glucose uptake than GLUT1 [35, 42]. Recently, GLUT4 has been shown to have a regulatory role during activity dependent increases in energy demand [43].

Cellular energy is produced through glycolysis (glucose to pyruvate) and oxidative metabolism (pyruvate to CO_2_) via the tricarboxylic acid (TCA) cycle and the electron transport chain. Glycolysis converts glucose to pyruvate in the cytosol in a series of enzymatic reactions generating adenosine triphosphate (ATP) and nicotinamide adenine dinucleotide (NADH). Briefly, glucose is converted into glucose 6-phosphate by hexokinase, a regulatory enzyme in glycolysis. Following the conversion of glucose to glucose 6-phosphate, glucose 6-phosphate continues through the glycolytic pathway or enters into the PPP. The PPP has two phases: oxidative and non-oxidative. The oxidative phase converts glucose-6-phosphate into ribulose-5-phosphate, CO_2_ and NADPH; the latter is important for maintaining redox balance. On the other hand, the non-oxidative phase gives rise to fructose 6-phosphate and glyceraldehyde 3-phosphate, as well as ribose 5-phosphate and xylulose-5-phosphate. The latter are important for the nucleotide biosynthesis of nucleic acids and sugar phosphate precursors for amino acid synthesis (reviewed in [44]). Glucose 6-phosphate can also be converted to glucose 1-phosphate to produce glycogen, an energy reserve mainly stored in astrocytes. Via glycolysis, glucose 6-phosphate forms fructose 6-phosphate catalyzed by phosphoglucomutase and later fructose 1,6-bisphosphate by another regulatory enzyme phosphofructokinase [45]. Fructose 1,6-bisphosphate gets converted into dihydroxyacetone phosphate, glyceraldehyde 3-phosphate, 2- and 3-phosphoglycerate, phosphoenol pyruvate and pyruvate by several enzymatic reactions producing NADH and ATP. Pyruvate can be converted into lactate by lactate dehydrogenase or into alanine by alanine aminotransferase. However, pyruvate largely enters the mitochondria and is converted to acetyl-CoA catalyzed by pyruvate dehydrogenase (PDH). In astrocytes, pyruvate can be carboxylated by pyruvate carboxylase to generate the TCA cycle intermediate oxaloacetate [46]. Within the TCA cycle a series of reactions oxidize acetyl-CoA and generate reducing equivalents, which then transfer electrons to oxygen via the enzyme complexes of the electron transport chain, ultimately resulting in the generation of ATP by ATP synthase also called complex V [47]. The TCA cycle is important not only in producing reduction equivalents for ATP generation, but it is also essential for providing intermediates to synthesize lipids, neurotransmitters and amino acids [34].

Energy generating processes and carbon metabolism are partially compartmentalized. In the CNS, there is cellular heterogeneity and different cells have different metabolic roles. The majority of cells in the CNS are neurons and astrocytes. Their metabolic cooperation in energy metabolism has been extensively studied. Each cell has different enzymes and transporters giving them unique metabolic characteristics. Astrocytes express pyruvate carboxylase [46] which converts pyruvate into oxaloacetate to replenish lost TCA cycle intermediates used to synthesize lipids, amino acids and neurotransmitters, including glutamate and glutamine. The majority of synapses in the CNS are glutamatergic [48]. During neurotransmission, neurons release glutamate, mostly synthetized from glucose, into the synaptic cleft. Glutamate is then taken up by astrocytes via glutamate transporters [49] and an astrocyte specific glutamine synthetase converts glutamate into glutamine [50]. Glutamine can be transferred to neurons and converted back to glutamate by phosphate activated glutaminase to complete the glutamate-glutamine cycle [51]. This cycle is important to protect neurons from excitotoxicity.

Glutamate can also be used as a substrate for oxidative metabolism. Seventy percent of glutamate in astrocytes is oxidatively metabolized by the TCA cycle, while 30% is converted into glutamine [52]. Similarly, GABA released from the synaptic cleft during neurotransmission can be taken up by astrocytes and enter the astrocytic TCA cycle as succinate via GABA transaminase and succinate semialdehyde dehydrogenase. Succinate can be further metabolized in the TCA cycle to form α-ketoglutarate, glutamate and subsequently glutamine. Astrocytes release glutamine where it is taken up by neurons to synthesize glutamate or GABA. In GABAergic cells, glutamate produced via phosphate activated glutaminase is decarboxylated to GABA. This glutamate/GABA-glutamine cycle is important to maintain the neurotransmitter pool in the CNS [53, 54].

Abnormalities in any step of these glucose metabolism pathways can result in reduced generation of ATP, which may affect basic cellular functions. Also, a decline in ATP levels can lead to a compensatory activation of other energy generating pathways, which may increase production of reactive oxygen species (ROS) and oxidative stress. In addition, since glucose metabolism is linked with amino acid neurotransmitter metabolism, abnormalities in glucose metabolism pathways may contribute to glutamate excitotoxicity and neurodegeneration.

## Disturbed energy homeostasis in patients with ALS

Energy homeostasis is the balance between energy intake and expenditure. This balance is maintained when uptake of nutrients is matched with energy expenditure, including thermoregulation, basal and physical activity. In patients with ALS, energy homeostasis has been shown to be disturbed [21, 55]. This is linked with increased resting energy expenditure (hypermetabolism) and/or decreased food intake [56, 57].

Hypermetabolism is a hallmark of many patients with ALS. Several reports have demonstrated that about 25–68% patients with ALS have increased resting energy expenditures [56–62]. In a study by Desport et al. [61], the resting energy expenditure of patients with ALS was on average 10% higher than healthy controls. Recently, hypermetabolism has been associated with significant reduction in function and reduced survival in patients [62]. Although various studies have suggested that hypermetabolism could lead to loss of body weight in patients with ALS, a recent study by Steyn et al. [62] reported no significant differences in body weight between hypermetabolic and normometabolic ALS patients, suggesting that other mechanisms such as loss of appetite and malnutrition may contribute to body weight loss. Indeed, loss of appetite has been shown in a subset of patients with ALS, which contributes to reduced food intake [63–65]. This is in agreement with studies that show malnutrition in patients with ALS. A study by Kasarskis et al. [59] showed that patients with ALS consumed 84% of recommended daily calories and are profoundly malnourished. Malnutrition is usually associated with dysphagia as a result of a weakness in bulbar muscles [66]. Moreover, malnourished patients with ALS have an average eightfold increased risk of death [67] and malnutrition overall is associated with poor survival [58].

Maintaining body weight in ALS is very important as it has been shown to affect ALS risk and survival [58, 68–70]. Compared to people with normal body weight, obese people were found to have 30–40% reduced risk of ALS [69]. In addition, reduced body mass index (BMI) is linked to poor survival outcome in patients with ALS [58, 68, 71]. For each 5% loss in body weight, Marin et al. [58] found a 30% increased risk of death. According to Paganoni et al. [68], the association between BMI and mortality is “U”-shaped, where patients with BMI 30–35 kg/m^2^ have the highest survival. On the other hand, BMIs < 18 and > 35 are associated with increased mortality. A recent study in large number of patients with ALS (n = 2420) demonstrated that more than two-thirds of patients exhibited weight loss at diagnosis. This was observed in 71.8% of bulbar-onset and 64.2% of spinal-onset patients with ALS. In addition, they found that for every 10% loss in body weight, there was a 23% increase in risk of death [72]. Interestingly, about 35.1% of patients with ALS lose body weight in spite of lack of symptoms of dysphagia indicating that the causes of body weight loss are multifactorial [72, 73]. Therefore, in order to reduce or prevent body weight loss, individually tailored therapeutic interventions for each underlying factor are needed [72].

Overall, maintaining body weight or promoting body weight gain may be of significant benefit in patients with ALS who lose weight. Therefore, various studies have investigated nutritional interventions with the aim of delaying disease progression in ALS. In a randomized clinical trial in 24 patients with ALS, high caloric diets with either high carbohydrate or high fat contents were shown to delay disease and prevent body weight loss compared to an isocaloric control diet [74]. For these reasons, dietary interventions which improve energy balance and maintain body mass index have been recommended in ALS [58, 71]. On the other hand, a recent randomized clinical trial investigating the efficacy and tolerability of a high calorie fatty diet in 208 patients with ALS did not find significant differences on survival between ALS and control patients [75]. Interestingly, posthoc analysis showed that high calorie diets prolonged life and stabilized body weight in patients with fast progressing disease [75]. Overall, it appears that there is impaired energy homeostasis in patients with ALS, and disease modifying properties of metabolic treatments in ALS confirm the key role of energy metabolism in the pathogenesis of the disease. Despite these whole-body metabolic changes being observed in patients with ALS, the origin of metabolic dysregulation remains to be fully elucidated. However, a number of pre-clinical studies indicate that underlying bioenergetic impairments at the cellular level may contribute to metabolic dysfunctions in ALS.

## Defects in CNS glucose metabolism in ALS

**Fig. 1:**
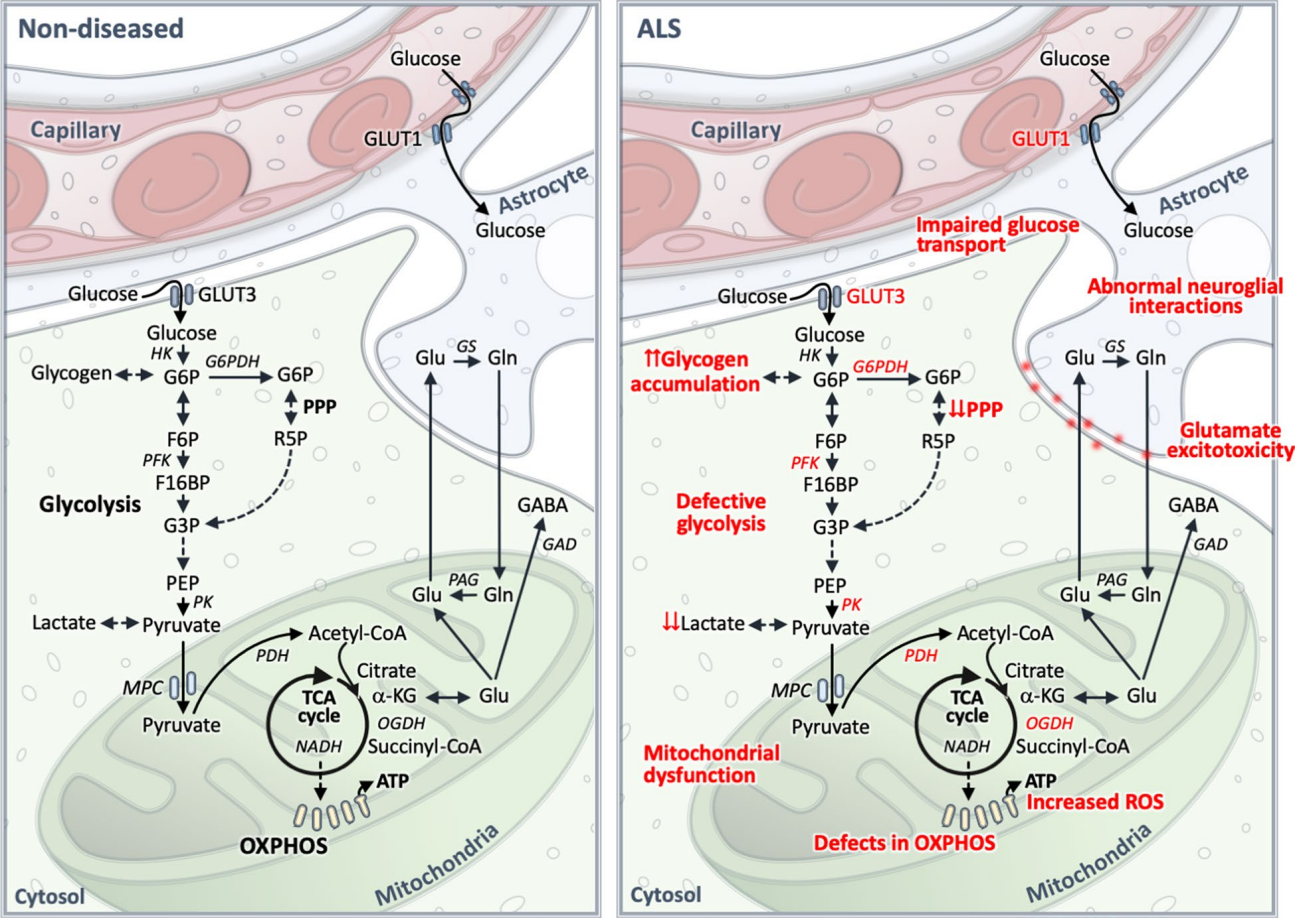
A simplified summary of CNS glucose metabolism, and commonly observed defects in amyotrophic lateral sclerosis (ALS). Glucose enters into neurons via glucose transporter (GLUT3) and via GLUT1 into astrocytes. In this figure, the astrocyte foot process is shown wrapping around the capillary. Then, glucose is phosphorylated by hexokinase (HK) to glucose 6-phosphate (G6P). G6P can be converted by glucose 6-phosphate dehydrogenase (G6PDH) to 6-phospho gluconolactone to enter the pentose phosphate pathway (PPP), where it is converted by a series of enzymatic reactions to subsequent PPP intermediates, such as R5P (ribose 5-phosphate) which later can enter the glycolytic pathway via glyceraldehyde 3-phosphate (G3P) or provide nucleotide backbones. G6P may also be converted to glucose 1-phosphate which is utilized for glycogen synthesis. If G6P continues through glycolysis, it is converted into F6P (fructose 6 phosphate) and later to fructose 1,6-bisphosphate (F16BP) by phosphofructokinase (PFK). F16BP is further metabolised to glyceraldehyde 3-phosphate (G3P), phosphoenol pyruvate (PEP) and pyruvate, by a series of enzymatic processes including pyruvate kinase (PK) which converts PEP into pyruvate. Pyruvate can be reduced to lactate by lactate dehydrogenase or enters mitochondria via mitochondrial pyruvate carrier (MPC) and gets converted into acetyl CoA by pyruvate dehydrogenase (PDH). Acetyl-CoA condenses with oxaloacetate to citrate and thereby enters the tricarboxylic acid (TCA) cycle. The TCA cycle generates different TCA cycle intermediates, including α-ketoglutarate (α-KG) from which glutamate can be synthesized. During neurotransmission, glutamate is released from the presynaptic vesicles into the synapse where it is taken up by glutamate transporters in astrocytes and then converted into glutamine (Gln) by glutamine synthetase (GS). Gln can also be transferred into neurons and gets converted into Glu by phosphate activated glutaminase (PAG), completing the Glu-Gln shuttle. In GABAergic neurons, Glu is converted into GABA by glutamate decarboxylase (GAD) enzyme. The TCA cycle also generates reducing equivalents such as nicotinamide adenine dinucleotide (NADH) and flavin adenine dinucleotide, which transfer electrons to oxygen via the enzyme complexes of the electron transport chain, ultimately resulting in the generation of ATP. In ALS, numerous metabolic defects (shown in red) at various steps in the glucose metabolism pathway that alter glucose metabolism and overall ATP generation have been described. This include impairments in glucose transport (changes in glucose transporter expression or HK activities), glycolysis (reduced activities of pyruvate kinase and phosphoglycerate kinase, reduced levels of lactate and reduced activities of PK), and PPP (reduced activities of G6PDH and reduced levels of R5P), increased glycogen accumulation, reduced entry of pyruvate into the TCA cycle (increased protein levels of PDH kinase 1, which downregulates PDH activity; reduced activities of oxoglutarate dehydrogenase (OGDH)), mitochondrial dysfunction, reduced mitochondrial ATP production, increased reactive oxygen species (ROS) production, as well as abnormal neuronal-glial interactions (reduced transfer of glutamate to glutamine, and glutamate excitotoxicity)

Various studies have shown changes in glucose utilization in certain brain and spinal cord regions of ALS patients as well as animal models of the disease. In addition, defects in specific energy generating metabolic pathways have been reported, which we discuss in the next sections. A simplified summary of CNS glucose metabolism, and commonly observed defects in ALS are shown in Fig. 1.

### Alterations in glucose transport or utilization

The most common technique used to quantify local cerebral glucose utilization (CMR_GLC_) is positron emission tomography (PET) with the radioactive glucose analogue ^18^fluoro-2-deoxyglucose (FDG). Like glucose, FDG is taken up by the brain via facilitative glucose transporters and is phosphorylated by hexokinase. Then FDG-6-phosphate is trapped as it cannot further be metabolized by glycolysis. The amount of radiation measured in the CNS during PET is thought to be indicative of the rate of glycolysis, because glucose transport through facilitative glucose transporters is unlimited and hexokinase is the rate limiting enzyme for glycolysis [76]. Therefore, low signals in FDG-PET (or low signals obtained with related methods using ^14^C 2-deoxy glucose autoradiography) are interpreted as low glucose utilization and/or hypometabolism. Here, it is important to note that although many studies use the terms ‘glucose metabolism’ and ‘glucose uptake’ interchangeably, imaging studies with ^18^F-FDG PET and ^14^C autoradiography can only indicate changes in glucose transport from the blood into brain, or in the first step in the glycolytic pathway, that is the rate of conversion of glucose into glucose 6 phosphate by hexokinase [77, 78].

Various PET and autoradiography studies with ^18^F and ^14^C 2-deoxy glucose have indicated widespread glucose hypometabolism in several brain regions in the motor cortex of patients with ALS [79–86]. The reason for this glucose hypometabolism is not clear as it was observed independent of cerebral atrophy (neuronal death) and old age [79, 80]. By contrast, increased FDG-PET signals in other brain regions including the amygdala, midbrain, and cerebellum [83–85, 87–89] as well as in the spinal cord [90, 91] have been ascribed to local activation of astrocytes and microglia [87, 89, 91]. Most of the earlier studies were done in small number of patients, and studies with larger numbers of patients with ALS have also shown similar glucose hypometabolism in frontal areas and hypermetabolism in the midbrain and cerebellum [88, 89]. Notably, using FDG-PET as a biomarker to discriminate patients with ALS from healthy controls, Pagani et al. [89] compared 195 patients with ALS to 40 healthy controls, and found glucose hypometabolism in the frontal and occipital cortex, and hypermetabolism in the midbrain of ALS patients. To consider the functional differences between spinal and bulbar onset ALS, Cistaro et al. [87] performed FDG-PET in 32 patients with ALS and 22 healthy subjects. They found relative decreases in signal in prefrontal, frontal and parietal brain regions in patients with bulbar onset disease when compared to healthy controls and ALS subjects with spinal onset disease. Similar patterns of glucose hypometabolism have been observed in familial ALS patients with C9ORF72 mutations when compared to patients without C9ORF72 mutations, where FDG-PET signals were reduced in the thalamus, basal ganglia and limbic cortex, while increased glucose utilization was observed in the midbrain [83, 92]. Interestingly, studies have linked glucose hypometabolism with disease progression [79, 86, 92–94]. Dalakas et al. [95] found a correlation between a decline in FDG signal and progressive weakness and upper motor neuron dysfunction. Likewise, the extent of hypometabolic/hypermetabolic spread in the brain was associated with disease progression in patients with ALS [86]. Also, widespread prefrontal glucose hypometabolism was linked with reduced ALS functional rating scale score (worsening of clinical symptoms) and shorter survival [92, 94].

Defective glucose metabolism has also been shown in the cortex and spinal cord of SOD1 mouse models of ALS [26, 96]. Reduced glucose utilization before the onset of symptoms (60 days of age) was observed in motor regions of the cerebral cortex of mutant SOD1^G93A^ mice and this hypometabolism extended to the spinal cord at 120 days of age [26]. In the spinal cord, glucose metabolism appeared to be reduced at early symptomatic and end stages of the disease in SOD1^G93A^ mice, but was increased before disease onset [96]. Recently, Weerasekera et al. used simultaneous ^18^F-FDG PET-magnetic resonance imaging and showed that glucose metabolism is decreased in the motor and somatosensory cortices of TDP-43^A315T^ mice while it was increased in midbrain region between 3 and 7 months of age, as compared to WT controls [97]. This suggests that similar changes in glucose metabolism may be observed across ALS disease models.

Although altered CNS glucose metabolism is a persistent feature in both human and animal models of ALS, the reasons why certain brain regions are hypometabolic while others are hypermetabolic are not clearly understood. Possible mechanisms for these changes may include reduced cerebral blood flow, and/or defects in glucose transport mechanisms or hexokinase activities. Reductions in cerebral blood flow that may affect provision of energy substrates to the brain have been shown in some CNS regions in animal models and patients with ALS [96, 98, 99]. Moreover, reduced expression of GLUT1 in the endothelial cells of lumbar and cervical spinal cord in SOD1^G93A^ mice indicate that glucose uptake could be decreased as lowered GLUT1 expression may limit glucose transport [100]. Indeed, reduced glucose transport has been observed in synaptosomes obtained from the cortex of SOD1^G93A^ mice [101]. In mutant SOD1^G37R^ mice, misfolded mutant SOD1 binds directly to the voltage dependent anion channel located on the outer mitochondrial membrane, thereby reducing hexokinase binding to the channel [102]. The consequences for reduced hexokinase binding are unknown. We have previously found no changes in hexokinase activity in cytosolic homogenates from the cortex and spinal cord of SOD1^G93A^ mice at the onset of disease [103]. By contrast, elevated hexokinase activities have been shown in synaptosomes obtained from the spinal cord of presymptomatic and symptomatic SOD1^G93A^ mice, as well cerebral cortex of symptomatic SOD1^G93A^ mice [104]. These differences may suggest that local glucose metabolism could be compartmentalized, and that different cellular compartments show different metabolic changes.

In general, most studies using glucose analogues in the CNS (particularly in the cortex) suggest that the ability of cells to take up glucose is impaired. Taken together, the mechanisms behind CNS hypometabolism and hypermetabolism are still unclear. Reduced neuronal cell density, reduced blood flow and reduced glucose transporter expression could in part explain glucose hypometabolism, while activation of glial cells was associated with hypermetabolism [87, 89]. Future studies are needed to identify mechanisms that underlie hypo- and/or hypermetabolism in the CNS of patients with ALS as well as animal models.

### Dysfunctions in glucose metabolism pathways in ALS

#### Glycolysis

Glucose partially metabolizes glucose to pyruvate, each lactate resulting in two molecules of ATP and NADH. Neurons uses glycolysis as a fast mechanism to generate ATP from glucose, particularly when there is an increase in energy demand nerve terminals rely heavily on glycolysis for synaptic function [105]. Glycolysis is also important for fast axonal transport [106]. In addition, during energy stress, a local increase in glycolytic enzyme clustering in presynaptic nerve terminals has been observed, indicating the importance of glycolysis to meet local energy demand [107].

In ALS, there is an increasing number of reports of defective glycolysis. We recently measured metabolite levels following injection of [1-^13^C]glucose in the CNS of wild type and SOD1^G93A^ mice at the onset and mid-symptomatic stage of disease using high pressure liquid chromatography, ^1^H and ^13^C nuclear magnetic resonance spectroscopy. We found a reduction in the levels of the total and ^13^C-glucose derived glycolytic metabolites lactate and alanine, but not glucose [108]. In a subsequent study where assessment was limited to the onset stage of disease, we found reductions in pyruvate levels and reduced activity of pyruvate kinase in cerebral cortex. However, no changes in hexokinase or pyruvate dehydrogenase activity, neither any changes in total amounts of other glycolytic and TCA cycle intermediates were observed [103]. Taken together, in CNS tissue of symptomatic SOD1^G93A^ mice, impairments in pyruvate production and conversion toward lactate and alanine were seen, which are indicative of defects in the glycolytic pathway. It is known that defective glycolysis can reduce entry of pyruvate into the TCA cycle and contribute to insufficient mitochondrial energy generation, thereby leading to a reduction in the ability to maintain basic cellular functions and capacity to counteract oxidative stress. In the cortex and spinal cord of symptomatic SOD1^G93A^ mice, diminished levels of some glycolysis-derived metabolites and less incorporation of ^13^C derived from ^13^C-glucose into TCA cycle derived neurotransmitters indicate impaired glucose carbon handling. In line with this, we have previously observed that in the CNS of SOD1^G93A^ mice, the first turn TCA cycle metabolites derived from [1-^13^C]glucose, including [4-^13^C]glutamate, [4-^13^C]glutamine and [2-^13^C]GABA were significantly decreased at mid-symptomatic stages of disease. This can be explained by reduced glucose entry into the TCA cycle as well as impairments in glutamate production from α-ketoglutarate [108]. In agreement with defective glycolysis, decreased gene expression of the glycolytic enzyme phosphoglycerate kinase 1, in SOD1^G93A^ mouse spinal cord has been observed [109]. In contrast to observations of glucose hypometabolism, increased expression of the glycolytic enzymes hexokinase, pyruvate kinase, and PDH kinase 1 was found in the motor neuron NSC-34 cell line [110]. Also, in a TDP-43 fly model, increased glycolytic activation has been reported [111]. In this regard, increased expression of glycolytic activities may signify an adaptive response for increased energy requirements in this disease.

Although little emphasis has been given to CNS glycolysis research in ALS, some studies indicate that activating the glycolytic pathway has the potential to modify ALS. Restoring NAD^+^ levels with supplementation of nicotinamide, an electron acceptor required to sustain glycolysis, was shown to modestly improve survival in the SOD1^G93A^ mouse model of ALS and increase glycolytic ATP production [43, 112]. In addition, a combination of two compounds that elevate NAD^+^ levels were found to improve ALS functional rating scale score, pulmonary function, and muscular strength in a small number of patients [113]. Likewise, activation of glycolysis via GLUT3 and phosphofructokinase overexpression in motor neurons has been found to be neuroprotective and improve locomotion in a TDP-43 *Drosophila* model, suggesting that glycolysis activators could slow disease progression and prolong survival in ALS [111]. Whether targeting of the glycolytic pathway would exert similar benefits in other models or in human ALS is an area of research that requires more investigation in future.

#### Glycogen metabolism

Glycogen is mainly stored in astrocytes and is rapidly used as a fuel source during brain stimulation. Glycogen breakdown directly yields glucose-6-phosphate; this can lead to the initiation of glycolysis with lower ATP requirement when compared to glucose, which requires one ATP molecule for phosphorylation by hexokinase to glucose-6-phosphate. Changes in the degradation of glycogen, or increased accumulation of glycogen could contribute towards changes in glycolytic pathways in ALS. Recently, Li et al. [114] reported increased levels of glycogen in the lumbar spinal cord of SOD1^G93A^ mice at the onset and end stage of disease, indicating that impairments in glycogen metabolism that occur upon the presentation of disease phenotype are maintained throughout the course of disease. In addition, in the spinal cord, the protein and mRNA levels of glycogen synthase (the enzyme that synthesizes glycogen) were unchanged, while those of phosphorylase B (the enzyme that degrades glycogen) were decreased. This indicates that glycogen accumulation in the spinal cord is due to impairments in glycogenolysis rather than increased glycogen synthesis (glycogenesis). Similarly, investigating metabolism in induced astrocytes from human familial ALS patients with C9ORF72 expansions, the mRNA and protein levels of glycogen mobilization enzymes glycogen phosphorylase and phosphoglucomutase were decreased [115], indicating reduced availability of energy from glycogen. In agreement with this, glycogen accumulation has also been reported in human ALS. Dodge et al. [116] found reduced neutral α-glucosidase activity (which degrades glycogen) in the spinal cord of late stage SOD1^G93A^ animals and in spinal cord tissue from people with ALS, confirming that glycogen accumulation is also a feature of human disease. Taken together, an altered ability to mobilise energy from glycogen stores is likely to affect energy metabolism in the spinal cord, but whether this contributes directly or indirectly to ALS pathology remains to be determined.

#### Pentose phosphate pathway (PPP)

The PPP is an important metabolic pathway which is directly linked to glycolysis, as glucose-6-phosphate, a metabolite produced through phosphorylation of glucose via hexokinase, is the substrate in both processes. The PPP is crucially needed to generate key pentose precursors for the synthesis of nucleotides and amino acids as well as NADPH for cellular defence against oxidative stress [44]. Around 5–6% of glucose is metabolized via PPP in neurons [117]. In the spinal cord of SOD1^G93A^ mice, we found a 35% reduction in total levels of ribose 5-phosphate together with threefold higher ^13^C enrichment in glucose-6-phosphate and a 15% decrease in glucose 6-phosphate dehydrogenase activity at onset of disease [103]. This indicates reduced PPP metabolism and reduced synthesis of ribose, rather than increased metabolism of ribose, which is also possible. Consistent with these findings in vivo, decreased mRNA expression and activities of the PPP enzymes glucose 6-phosphate dehydrogenase and 6-phosphogluconate dehydrogenase have been observed in mutant SOD1 transfected NSC-34 cells [118]. The PPP plays a major role in generating NADPH, which is required for glutathione maintenance in its reduced state and thereby protects neurons from oxidative stress. Glutathione is a major antioxidant molecule that detoxifies ROS in the brain. Reduced levels of NADPH [118], lower glutathione levels [119, 120] and increased oxidative stress are observed in ALS, corroborating that impairments in the PPP might further aggravate oxidative stress in the disease. In conclusion, an impaired PPP in ALS CNS tissue could contribute to an overall reduction in glucose utilisation as well as ribose and NADPH synthesis, resulting in irregularities with nucleotide synthesis and increased oxidative stress. Further research is needed to assess if impairments in PPP contribute to ALS pathology and if activation of the PPP could result in therapeutic benefit.

#### TCA cycle

Few changes in the TCA cycle have been reported in the CNS. In the motor cortex of patients with ALS, there was less mRNA expression of enzymes contributing to the TCA cycle, such as the cytosolic malate dehydrogenase 1 (an enzyme involved in metabolic cooperation between cytosol with mitochondria) [121]. In motor neuronal cultures obtained from SOD mice as well as in NSC-34 cells, protein levels of PDH kinase 1, which downregulates PDH activity, was increased, and this in turn is expected to reduce the entry of pyruvate into the TCA cycle [110, 120]. In addition, alterations in expression of enzymes involved in TCA cycle metabolism such as decreased mRNA levels of isocitrate dehydrogenase 3α (involved in TCA cycle metabolism) in astrocytes [122] and oxoglutarate dehydrogenase (a key enzyme that controls metabolic flux through the TCA cycle) in SOD1^G93A^ mice spinal cord have been reported [123]. In our own work, we have found reduced activity of oxoglutarate dehydrogenase in the cortex of SOD1^G93A^ mice [103]. Reduction in the activities of TCA cycle enzymes in CNS tissues in ALS could be partly explained by oxidative stress. As activities of TCA cycle enzymes are inhibited during increased cellular oxidative stress as a possible adaptive response against ROS and to provide antioxidant effects, inhibition of TCA cycle enzymes results in lower NADH generation, and diminished availability of electrons to enter the electron transport chain [124, 125].

#### Oxidative phosphorylation and mitochondrial dysfunctions

Mitochondria are the primary site of energy production. However, they are also the main sources of ROS. Uncontrolled production of ROS may damage lipids, proteins and/or nucleic acids and as a result, impair mitochondrial function [126]. In the spinal cord and brain of patients with ALS and in mouse models of ALS, several studies have shown functional and morphological impairments in mitochondria [127–129]. These defects may contribute to reduced energy production or increased oxidative stress. In addition, several defects in electron transport chain activities, reduced oxidative phosphorylation and subsequent generation of ATP have been reported [26, 127, 130–133]. Particularly, subunits of ATP synthase, the last enzyme complex at the end of mitochondrial oxidative phosphorylation responsible for ATP production from ADP, showed lower expression in spinal cord [134, 135] and motor cortex of patients with ALS [121], which may lead to reduced ATP generation. In contrast, in motor neurons isolated from pre-symptomatic SOD1^G93A^ mice, increased expression of genes involved in mitochondrial machinery including ATP synthase was found [122]. This could be due to an early compensatory activation of energy generating pathways to counteract increased energy demand. Overall, mitochondrial dysfunction and its physiological consequences can lead to depletion of cellular energy and subsequent death of motor neurons. Several excellent reviews have been published on mitochondrial abnormalities in ALS and we encourage the reader to access these resources for a more detailed overview [136–138].

Overall, the mechanisms underlying altered CNS glucose metabolism in ALS are not clearly understood and remain to be determined. However, possible mechanisms that can drive glucose metabolism abnormalities include mutant protein aggregation and oxidative stress. Studies have shown that mutant TDP-43 accumulates in the mitochondria in patients with ALS which leads to impairments in complex I activities causing defective cellular energetics [139]. In addition, *Drosophila* with mutant human FUS overexpression have been shown to have fragmented mitochondria because of pathological mitochondrial aggregation of FUS [140]. Both mutant TDP-43 and FUS have also been shown to impair mitochondrial- endoplasmic reticulum interactions leading to impaired protein homeostasis and reduced mitochondrial ATP production [141, 142]. Also, accumulation of mutant SOD1 protein has been found in brain mitochondria that caused defective complex activities in the electron transport chain particularly complex I and IV [143, 144]. These studies suggest that mitochondrial protein aggregation may cause structural and functional abnormalities in mitochondria leading to impaired glucose metabolism and generation of ATP.

Defective glucose metabolism in the CNS may also arise as a result of increased oxidative stress, an imbalance between ROS and antioxidant defence mechanisms. Numerous studies have shown that oxidative stress is involved in the onset and progression of ALS (reviewed in [19]). As such, oxidative damage to the enzymes involved in the glucose metabolism pathways as well as to mitochondrial DNA may lead to defective energy production. Indeed, studies have shown oxidative modifications of glycolytic, TCA cycle and mitochondrial proteins in the CNS in neurodegenerative disorders [145, 146]. This ultimately may lead to disrupted neuronal energy metabolism and worsening of oxidative stress. In summary, further studies are required to identify specific mechanisms underlying defective glucose metabolism in ALS and also to better understand the disease and find therapeutic targets.

## Defects in cellular energy metabolism: neurons, glia and their interactions

The progressive loss of motor neurons is the hallmark of ALS, however, it is acknowledged that non-neuronal cells, including astrocytes [147, 148] and oligodendrocytes [149–151] affect motor neuron death and disease progression. The metabolic interaction of neurons and astrocytes is crucial for limiting glutamate mediated excitotoxicity, providing metabolic substrates and in defending against oxidative stress (reviewed in [152]). In patients with ALS, abnormalities in neuronal and astrocytic interactions with regard to glutamate metabolism have been observed [15, 153, 154]. Several early reports have shown the loss of expression of the main glutamate transporter protein—1/ excitatory amino acid transporter—2 in the spinal cord [155–159] and the motor cortex [157] of patients with ALS or animal models, which can result in a decrease in glutamate transport. Indeed, reduced glutamate transport was shown in synaptosomes obtained from the brain and spinal cord tissues of patients with ALS and animal models [101, 154]. In SOD1^G93A^ mice spinal cord, we found reduced glutamate and glutamine production from glucose as well as reduced transfer of glutamate to glutamine [108]. Reduced glutamate uptake can contribute to excitotoxicity. Consistent with this, increasing EAAT2 expression to increase glutamate uptake into astrocytes has been shown to protect motor neurons from death and extend survival in mouse and cellular models of ALS [160, 161], while loss of its expression reduced survival of SOD1^G93A^ mice [162]. Riluzole, an approved drug in the treatment of ALS is believed to reduce glutamate neurotransmission by inhibiting glutamate release [163, 164], enhancing astrocytic uptake of glutamate by activating glutamate transporters [165–167] and inhibition of a persistent sodium current, resulting in less repetitive firing [168]. Overall, dysregulation of astrocytic and neuronal interactions may contribute to excitotoxicity and neurodegeneration.

Astrocytes and oligodendrocytes provide metabolic support to neurons. There is emerging evidence to suggest that during neurotransmission, glutamate uptake in astrocytes leads to activation of glycolysis and lactate production [169]. Lactate can then be released into extracellular fluid via the monocarboxylate transporters (MCT1 and MCT4) and can be transferred to neurons (via MCT2), where it can be utilized as an energy substrate. Lactate can be converted into pyruvate by lactate dehydrogenase and oxidized in the mitochondrial TCA cycle. In ALS, various studies have shown reduced expression of monocarboxylate transporters which may hinder glial metabolic support to neurons [109, 151, 170]. Decreased expressions of MCT1 and MCT4 in the motor cortex of patients with ALS, and reduced MCT1 expression in SOD1^G93A^ mouse spinal cord [151], may reduce lactate transport to neurons, and lactate transport from oligodendrocytes to neurons. In addition, when glucose availability is low, as is seen in cases of excess neuronal activity or diseases, astrocytes can use glycogen and thereby spare glucose for neurons [171]. Also, astrocytes are able to produce lactate from glycogen and may provide lactate or glutamine to neurons thereby preventing energy depletion [172, 173]. In ALS, however, the ability of astrocytes in providing metabolic support to neurons seems to be impaired as abnormalities in glycogen metabolism have been observed in ALS, please see the discussion above [114–116].

A recent study used a phenotypic metabolic array to profile fibroblasts and induced neuronal progenitor-derived human induced astrocytes from people with C9ORF72 ALS compared to normal controls to measure the rates of production of NADH from 91 potential energy substrates [115]. Distinct metabolic profiles were found in fibroblasts and reprogrammed induced astrocytes derived from C9ORF72 and sporadic patients with ALS when compared to control astrocytes. Mostly there was less metabolic flexibility in ALS astrocytes, which was apparent by impaired metabolism of pyruvate, galactose and succinate. In contrast, there were no changes in acetate metabolism in cortex and spinal cord of SOD1^G93A^ mice, indicating that the astrocyte TCA cycle is functional in ALS [108]. Also, there were impairments in adenosine, fructose and glycogen metabolism, as well as disruptions in the membrane transport of mitochondrial specific energy substrates [115]. Thus, the induced human ALS astrocytes starved more quickly when energy supply was limited. In a related report, C9ORF72 and sporadic ALS human induced astrocytes and fibroblasts were shown to have an adenosine to inosine deamination defect caused by reduction of adenosine deaminase [174]. This coincides with lower levels of inosine in the lumbar spinal cords of C57 SOD1^G93A^ mice at onset of disease, however in 129 transgenic mice and at later stages no alterations in inosine amounts were seen [175]. Patient-derived induced astrocyte lines and neurons in co-cultures were more susceptible to adenosine-induced toxicity, which could be mimicked by inhibiting adenosine deaminase in control lines [174]. Interestingly, adding inosine reduced toxicity and highlights that increasing inosine may be a potential therapeutic approach. Overall, given the defects in neuronal-glial interactions in ALS, improving glial cell metabolic support to neurons could be one potential therapeutic avenue in ALS, and further studies investigating this approach are needed.

## Targeting glucose metabolism in ALS

Currently, there is no effective treatment for ALS. Riluzole, and recently edaravone are the only approved drugs [176–178]. Riluzole improves life expectancy by 2–3 months [176, 179], although a recently study indicated that it may prolong survival by 6–19 months [180]. The exact mechanisms by which riluzole exerts these beneficial effects are not clearly known, however it is believed that it is due to a reduction in glutamatergic neurotransmission and inhibition of a persistent sodium current [163–167, 181]. Interestingly, some studies have shown other mechanisms not directly related to its action on glutamate and sodium channels. Riluzole was able to increase the rate of glucose transport by upregulating GLUT1 and GLUT3 transporters in NSC-34 cells [182]. It was also shown to improve intracellular glucose uptake in cortical areas in patients with Huntington’s disease [183]. Additionally, chronic riluzole treatment was shown to increase [1-^13^C]glucose metabolism and the glutamate-glutamine cycle in prefrontal cortex and hippocampus of rats [184]. These studies suggest that riluzole may have a role in improving CNS glucose metabolism. Similarly, edaravone, a free radical scavenger with antioxidant effects [185, 186] has been shown to improve brain glucose uptake in rats after injury [187]. While it is possible that riluzole and edaravone could in part modify disease progression through their actions on glucose metabolism, further studies are required to fully elucidate this potential mechanism of action in ALS.

Although little attention is given to metabolic therapies in ALS, targeting glucose metabolism has the potential to slow the onset and progression of ALS. Compounds that enhance glucose uptake and its metabolism via glycolysis and PPP could be beneficial by improving ATP generation and reducing oxidative stress. Interestingly, glycolysis activation has been shown to reduce disease progression in other neurodegenerative disorders such as Parkinson’s disease [188]. Recently, Manzo et al. showed that upregulation of glycolysis by increasing motor neuron expression of GLUT3 and phosphofructokinase in TDP-43 ALS models is neuroprotective [111]. Improving glucose metabolism not only provides ATP, but it may also have additional benefits in reducing abnormal protein aggregation and reducing oxidative stress; two other mechanisms hypothesized to drive the onset and progression of ALS. ATP has been found to have a biological hydrotope property (an amphiphilic molecule that causes solubilization of hydrophobic compounds) [189]. Thus, a higher concentration of ATP may prevent the formation of protein aggregates and also has the capacity to dissolve already formed protein aggregates [189]. These investigations signify that metabolic therapy particularly those targeting glycolysis and PPP is a worthwhile approach to delay disease progression and improve survival in people with ALS as well as other neurodegenerative disorders.

**Fig. 2:**
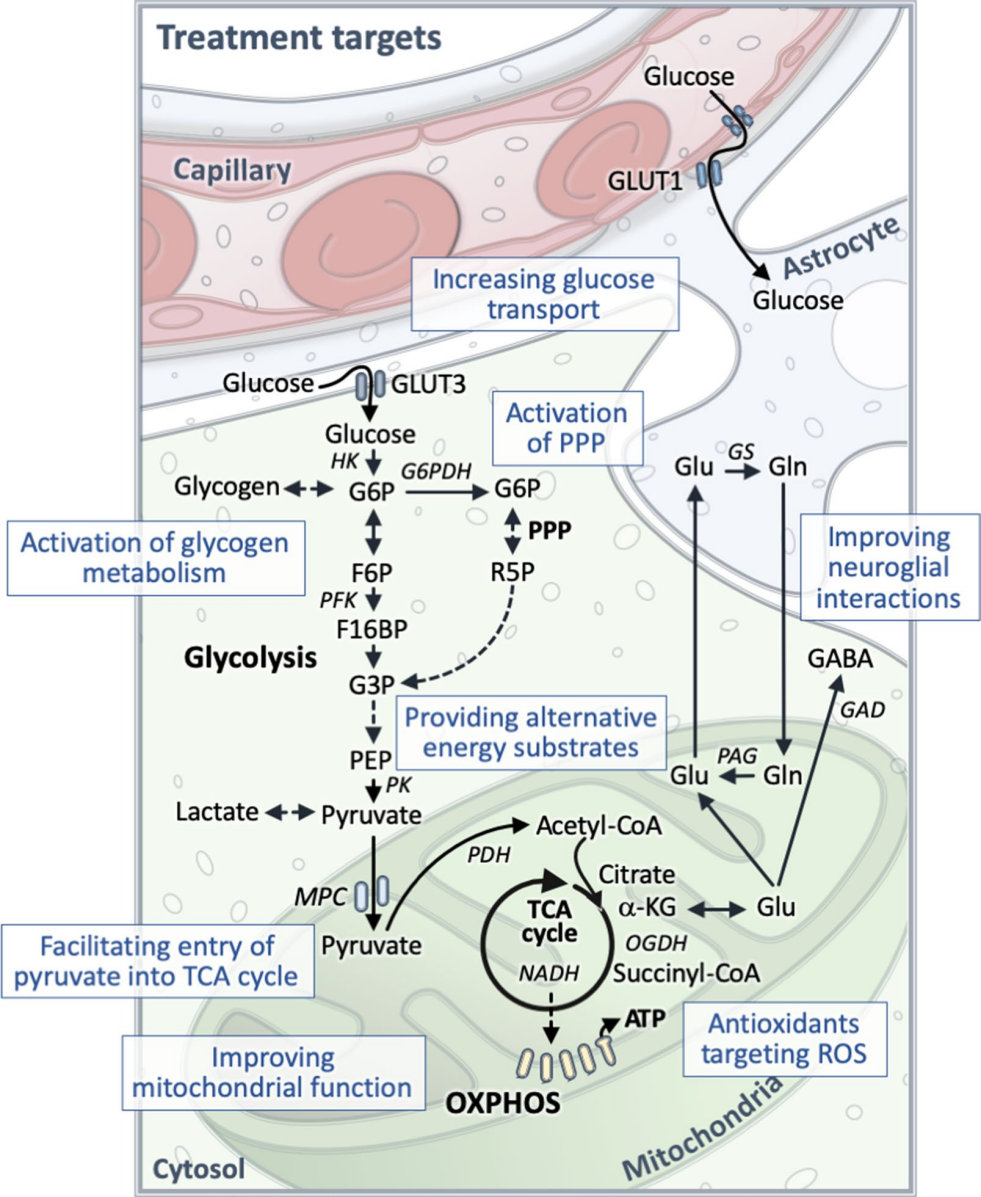
Targeting of CNS glucose metabolism in amyotrophic lateral sclerosis (ALS). Targeting CNS glucose metabolism has the potential to reduce the onset and progression of ALS. Possible approaches or targets in the glucose metabolism pathway include activation of glucose transport, glycolysis, pentose phosphate pathway (PPP), and pyruvate entry into the TCA cycle, facilitation of glycogen degradation, provision of alternative energy substrates, improving oxidative phosphorylation (OXPHOS) and ATP generation, using antioxidants targeting mitochondrial reactive oxygen species (ROS) as well as improving overall mitochondrial function and neuroglial interactions

Alternative and anaplerotic metabolic substrates that refill lost TCA cycle intermediates could also be used to improve energy supply in ALS. In bypassing the rate limiting steps that are linked with impaired neuronal glucose metabolism, such substrates can restore mitochondrial ATP production [190]. Metabolic treatments that either provide high amounts of energy, alternative substrates to glucose, or that can increase TCA cycling or mitochondrial function have been investigated in ALS. These include the ketogenic diet, caprylic triglyceride, the “Deanna protocol”, dichloroacetate, pyruvate, lactate, and triheptanoin (previously reviewed in [22, 24, 191]). Although the outcomes from in vitro and in vivo studies of metabolic treatments were positive to varying degrees, further investigations in patients with ALS are needed to inform whether these outcomes can be translated in humans. Studies with energy substrates including different combinations are also worth investigating as a means to slow the disease process and improve quality of life. Also, it will be important to find treatments that decrease oxidative stress, since metabolic abnormalities are strongly linked to oxidative stress. Particularly, treatment of ALS with antioxidants targeted against mitochondrial ROS together with metabolic energy substrates could result in better treatment outcomes. Previously, oxidative stress has been shown to be counteracted by supplying energy substrates, including pyruvate, acetoacetate, triheptanoin and β–hydroxybutyrate, which are all metabolized by the TCA cycle [192–195]. Further research investigating the role of metabolic fuels in combination with antioxidants or other treatments in delaying disease progression is needed. A summary of how glucose metabolism can be targeted in ALS is shown in Fig. 2.

## Conclusions

Whole body metabolic changes are consistently observed in animal models of ALS as well as patients with ALS. In CNS tissues, defects in energy producing pathways are common. Among the common perturbations are a decrease in glucose uptake, and impairments in glycolysis and the PPP. In addition, abnormalities in mitochondrial function and ATP generation have been observed. Compounds that activate glucose metabolism, particularly via glycolysis, and the PPP may have the potential to slow disease progression. Although various investigations targeting glucose metabolism have shown beneficial outcomes in animal models, to which extent this translates to effective therapies in humans remains to be confirmed. Given the heterogeneity of disease, metabolic changes in patients with ALS are also likely to be highly variable. Therefore, individually tailored treatments may be needed to achieve the best outcomes for people living with ALS. This is an area of research that warrants further investigation.

## Data Availability

Not applicable.

## Abbreviations

ALS: Amyotrophic lateral sclerosis
BMI: Body mass index
C9ORF72: Chromosome 9 open reading frame 72
F16BP: Fructose 1,6-bisphosphate
F6P: Fructose 6-phosphate
FDG—PET: Fluoro deoxyglucose—positron emission tomography
FUS: Fused in sarcoma
G3P: Glyceraldehyde 3-phosphate
G6P: Glucose 6-phosphate
G6PDH: Glucose 6-phosphate dehydrogenase
GABA: γ-Aminobutyric acid
GLUT: Glucose transporter
GS: Glutamine synthetase
HK: Hexokinase
α-KG: α-Ketoglutarate
MCT: Monocarboxylate transporter
MPC: Mitochondrial pyruvate carrier
OGDH: Oxoglutarate dehydrogenase
OXPHOS: Oxidative phosphorylation
PAG: Phosphate activated glutaminase
PDH: Pyruvate dehydrogenase
PEP: Phosphoenol pyruvate
PFK: Phosphofructokinase
PK: Pyruvate kinase
PPP: Pentose phosphate pathway
R5P: Ribose 5-phosphate
ROS: Reactive oxygen species
SOD: Superoxide dismutase
TDP-43: TAR DNA-Binding Protein-43
